# A systematic approach for developing mechanistic models for realistic simulation of cancer cell motion and deformation

**DOI:** 10.1038/s41598-021-00905-3

**Published:** 2021-11-03

**Authors:** Pouyan Keshavarz Motamed, Nima Maftoon

**Affiliations:** 1 Department of Systems Design Engineering, University of Waterloo, 200 University Avenue West, Waterloo, ON N2L 3G1 Canada; 2 Centre for Bioengineering and Biotechnology, University of Waterloo, Waterloo, ON Canada

**Keywords:** Computational biophysics, Cancer models, Biomedical engineering

## Abstract

Understanding and predicting metastatic progression and developing novel diagnostic methods can highly benefit from accurate models of the deformability of cancer cells. Spring-based network models of cells can provide a versatile way of integrating deforming cancer cells with other physical and biochemical phenomena, but these models have parameters that need to be accurately identified. In this study we established a systematic method for identifying parameters of spring-network models of cancer cells. We developed a genetic algorithm and coupled it to the fluid–solid interaction model of the cell, immersed in blood plasma or other fluids, to minimize the difference between numerical and experimental data of cell motion and deformation. We used the method to create a validated model for the human lung cancer cell line (H1975), employing existing experimental data of its deformation in a narrow microchannel constriction considering cell-wall friction. Furthermore, using this validated model with accurately identified parameters, we studied the details of motion and deformation of the cancer cell in the microchannel constriction and the effects of flow rates on them. We found that ignoring the viscosity of the cell membrane and the friction between the cell and wall can introduce remarkable errors.

## Introduction

Metastasis is the most lethal cause of cancer-associated deaths^1^. The metastasis cascade includes cancerous cells detachment from the primary tumor, intravasation into the circulatory or lymphatic system, traveling in the body, extravasation, and growth in a secondary site. The cancer cells that intravasate and enter the bloodstream are known as circulating tumor cells (CTCs). The deformability of CTCs plays an important role in the metastasis cascade because entrapment of CTCs in capillaries and their penetration into the endothelial cell–cell junctions during intravasation and extravasation highly depend on their drastic deformation^2^. Therefore, developing novel diagnostic tools and novel tools for predicting metastatic spreading requires accurate quantitative models of cell deformability. Such models require experimental data of cancer cell deformation as well as highly efficient computational methods in terms of calculation time.

The difference between the deformability of healthy and diseased cells motivated several experimental studies to find label-free biomarkers for differentiating these cells using techniques such as optical tweezers^3^, atomic force microscopy^4^, and micropipette aspiration^5^. Furthermore, the recent progress in microfabrication techniques has helped microfluidic devices to become the ultimate choice in studying cell behaviour^6^ thanks to their quick and high throughput operation. Microfluidic devices were also made for capturing and sorting cancer cells based on cell deformability^7^. In one type of these microfluidic devices, a constricted microchannel is used to study the deformability of cells in terms of the time required for the cell to deform and pass through the constriction. Because experiments revealed that the more malignant the cells, the more deformable they are, the passage time of cancer cells through the constricted microchannel was sought as a good index for cell malignancy^8–10^.

Because computational models can be used to design and improve such microfluidic devices, the development of models of cells which are suspended in fluid, and deform to enter and pass through a narrow channel has attracted many researchers^11–14^. The numerical methods used for modelling of motion and deformation of soft materials suspended in a fluid domain are categorized into continuum and discrete methods^15^ and were reviewed previously^16^. In continuum approach, mainly, (visco)elastic solid models^17,18^ and liquid drop models^12,14,19^ were used in order to simulate the deformation of cells. However, the continuum solid models are computationally expensive for modelling deformations of cells in interaction with the surrounding fluid domain and the liquid drop models are insufficient in simulating cell transit in the constriction since they assume a lubrication layer between the cell and the constriction walls^14^.

Complexity of interactions at the cellular level motivated researchers to use discrete methods in the recent years^20^. In discrete methods, the fluid phase is modelled using various methods such as dissipative particle dynamics^21^, smoothed particle hydrodynamic^22^, and lattice Boltzmann method (LBM)^23,24^. LBM has become popular due to its intrinsic parallelization ability and acceptable accuracy in modelling fluid flow in microcapillaries with low Reynolds numbers^25^. In discrete methods, fluid-cell interactions are modelled using immersed boundary method^24,26^ or dissipative coupling method (DCM)^23^. Furthermore, in discrete methods, the cell membrane is discretized by a triangular spring network as a coarse-grained spectrin-link model^21,27^. The computational efficiency of spring-based network models over continuum models of red blood cells (RBCs) suspended in fluid was demonestrated^28^ and many authors used these models to study motion of deforming cells in fluid flow. For example, Cimrák et al. developed a spring-network model for describing the flow of cells in microchannels^23^. Xiao et al.^11^ modelled the cell deformation passing a narrow slit using a discrete method in which the flow was modelled using dissipative particle dynamics, while Tan et al.^29^ modelled the fluid domain with LBM and conducted a parametric study on the effects of various micropore size and the applied pressure on the squeezing of a CTC through micropores.

Because discrete spring-based network models are efficient for modelling cell deformation in terms of accuracy and computational cost, they can be scaled up to organ-level models of metastasis that include circulation, entrapment, and extravasation of CTCs. They can also be used for developing and optimizing microfluidic devices for cancer diagnosis. The spring-network models include a few parameters which represent the viscoelastic material properties of the cell in discrete domain^30^ and should be identified for each cell type, cell size, and mesh arrangement through a lengthy process to correctly reproduce experimental measurements. There have been studies that identified spring-network parameters for RBCs^15,24^. However, to the best of our knowledge, there were no studies that identified spring-network parameters for cancer cells and for other cell types the spring-network parameters were identified through manual adjustments. There has not been any structured method for identifying the spring-network model parameters by minimizing the error between model calculations and experimental data.

In this paper, to identify the parameters of the spring-network model of cancer cells, we developed a method by coupling an optimization algorithm to a discrete model of the cancer cell (human lung cancer cell) passing through a constriction. This model considered friction between the cancer cell and the microchannel wall. The optimization was performed using genetic algorithm (GA) and existing experimental data of deformation of cancer cells in a microfluidic constriction was employed^9^. In our model, in addition to the spring-network model for the deformation and motion of the cell, the fluid flow was modelled using LBM, and dissipative coupling was used to model interactions between the cell and fluid flow. This study can be a corner stone for developing numerical models related to: (a) cancer-cell deformations in microcapillaries, (b) finding cancer cell material properties^31^, and (c) designing advanced microfluidic devices for deformability-based cancer diagnosis.

## Materials and methods

### Lattice Boltzmann method and dissipative coupling

All computational simulations were performed in Extensible Simulation Package for Research on Soft Matter (ESPResSo) open-source code (version 4.0.0)^32^ using its Object-in-Fluid (OIF) module^33^ for modelling viscoelastic cells interacting with fluid flow and microchannel walls. Customarily, at the macroscopic level, fluid flow is treated as a continuum and analyzed by solving the Navier–Stokes equations. However, in recent years, LBM became a powerful method of choice, especially at the cellular length scale, due to its high calculation efficiency and its intrinsic parallelism^15^. In LBM, the fluid flow is discretized in time and space by considering the fluid as fictitious particles. In the present work, three-dimensional 19-velocity cube lattice scheme (D3Q19) has been utilized in the LBM governing equations as follows:

1 $${n}_{i}\left(x+{\delta }_{x}, t+{\delta }_{t}\right)={n}_{i}\left(x,t\right)-\frac{1}{\tau }\left({n}_{i}\left(x,t\right)-{{n}_{i}}^{eq}\left(x,t\right)\right)+{f}_{i}\left(x,t\right) \, for \, i=1, 2, \dots , 19$$

where ${n}_{i}\left(x,t\right)$, ${\delta }_{t}$, ${\delta }_{x}$, $\tau$ and ${f}_{i}\left(x,t\right)$ are the density distribution function, time step, grid spacing, relaxation time toward the equilibrium distribution ${{n}_{i}}^{eq}$, and external force, respectively. At each lattice site, the macroscopic fluid density *ρ* and velocity *u* can be obtained from the particle density functions as follows:

2 $$\rho \left(x,t\right)=\sum_{i}{n}_{i}\left(x,t\right) \, for \, i=1, 2, \dots , 19$$

3 $$\rho \left(x,t\right)u=\sum_{i}{n}_{i}\left(x,t\right){e}_{i} \, for \, i=1, 2, \dots , 19$$

where, ${e}_{i}$ is the discrete velocity vector^34^.

Immersed boundary nodes were defined using vertices of triangular mesh representing the surface of the cell membrane. Motions of these nodes due to fluid forces and viscoelastic properties of the cell membrane are governed by the Newton’s second law of motion:

4 $${m}_{j}\frac{{d}^{2}x}{{dt}^{2}}={F}_{j} \, for \, j=1, 2, \dots , number \, of \,  nodes$$

where *m*_*j*_ is the mass of the node *j,* and *F*_*j*_ is the force applied to that node. The viscoelastic behaviour of the cell is defined by elastic and viscous parameters which will be explained in “Spring-network model of cell”.

In the dissipative coupling method, the coupling are done by means of the drag force exerted by the fluid on the nodes of the cell membrane^33^ as follows

5 $${F}_{j}=\xi \left(v-u\right) \, for \, j=1, 2, \dots , number \, of \, nodes$$

where $v$ is the velocity of the cell-membrane node, and $u$ is the velocity of the fluid at the same position. In this equation, $\xi$ is the phenomenological friction coefficient between fluid and the immersed object that was already calibrated using experimental data^35^. It was reported that $\xi$ is dependent on the surface area of the immersed object ($S$), the number of cell membrane mesh nodes ($n$) and the dynamic viscosity of the fluid ($\upsilon$). Equation 6 defines $\xi$ for a sphere with radius *r* and discretized into $n$ nodes as follows:

6 $${\xi }_{n, r}=\frac{{n}_{ref}}{n}\frac{\sqrt{S}}{\sqrt{{S}_{ref}}}{\xi }_{ref}$$

where ${\xi }_{ref}$ is the calibrated friction coefficient for a reference sphere, given in Table 1, ${n}_{ref}$ is 393, and ${S}_{ref}$ is the reference surface area of the reference sphere with the reference radius ${r}_{ref}$ of 4 µm^35^.

**Table 1:** ${\xi }_{ref}$ for the reference sphere (*r* = 4 µm, *n* = 393) immersed in three different fluids^35^.

$\upsilon \left(mPa\cdot s\right)$	${\xi }_{ref} \left(nN\cdot \frac{s}{m}\right)$
1.5375	1.82
1.3	1.54
1.0	1.18

### Spring-network model of cell

We used the viscoelastic cell model implemented in OIF in which the cell is discretized employing a two-dimensional triangular network of parallel springs and dashpots according to the Kelvin-Voigt viscoelastic model. In this model, the deformation behaviour of the cell is imitated using one viscous damping coefficient and five elastic moduli (stretching, bending, local area, global area, and volume) as described below^15^.

#### Stretching modulus

The stretch of a spring spanning as an edge on the cell membrane mesh connecting cell membrane vertices A and B is defined as^23^:

7 $${\overrightarrow{F}}_{s}\left(A\right)={k}_{s}\kappa \left(\lambda \right)\frac{L-{L}_{0}}{{L}_{0}}{\overrightarrow{p}}_{AB}$$

where ${k}_{s}$, ${L}_{0}$, $L$, and ${\overrightarrow{p}}_{AB}$ are the stretching modulus, the relaxed length of the edge AB, the current length of the edge AB, and the unit vector pointing from vertex A to vertex B, respectively. Moreover, $\lambda =\frac{L}{{L}_{0}}$ is the stretch and $\kappa \left(\lambda \right)$ describes the neo-Hookean hyperelastic behaviour of the edge^36^:

8 $$\kappa \left(\lambda \right)=\frac{{\lambda }_{AB}^{0.5}+{\lambda }_{AB}^{-2.5}}{{\lambda }_{AB}+{\lambda }_{AB}^{-3}}$$

#### Bending modulus

Bending is defined in terms of the changes in the angles between two neighbouring triangles. For triangle ABC, the bending force can be calculated as follows^23^:

9 $${\overrightarrow{F}}_{b}\left(ABC\right)={k}_{b}\frac{\theta -{\theta }_{0}}{{\theta }_{0}}{\overrightarrow{n}}_{ABC}$$

where ${\theta }_{0}$ and $\theta$ are the angle between two triangles in the resting and current shapes, respectively. ${k}_{b}$ is the bending modulus and ${\overrightarrow{n}}_{ABC}$ is the unit vector normal to the triangle.

#### Local area, global area and global volume moduli

The biology of the cell membrane necessitates resisting to changes in the local area, global area and volume of the whole cell^15^. For node *A*, forces applied on each node of the triangle *ABC* for satisfying the local area, global area, and global volume preserving constraints can be computed from:

10 $${\overrightarrow{F}}_{a}\left(A\right)=-{k}_{al}\frac{{S}_{ABC}-{S}_{ABC}^{0}}{{t}_{a}^{2}+{t}_{b}^{2}+{t}_{c}^{2}}\overrightarrow{AT}$$

11 $${\overrightarrow{F}}_{ag}\left(A\right)=-{k}_{ag}\frac{S-{S}^{0}}{{S}^{0}}{S}_{ABC}\frac{\overrightarrow{AT}}{{t}_{a}^{2}+{t}_{b}^{2}+{t}_{c}^{2}}$$

12 $${\overrightarrow{F}}_{V}\left(A\right)=-{k}_{V}\frac{V-{V}_{0}}{{V}_{0}}{S}_{ABC}{\overrightarrow{n}}_{ABC}$$

where ${S}_{ABC}^{0}$, ${S}_{ABC}$ are the areas of the triangle *ABC* in the resting and current shapes, ${t}_{a}$, ${t}_{b}$, ${t}_{c}$ are the distances of the triangle vertices to its centroid T. ${S}^{0}$ and $S$ are the areas of the entire cell in resting and current shapes, ${V}_{0}$ and $V$ are the whole volumes of cell in the resting and current shapes. Also, ${k}_{al}$, ${k}_{ag}$, and ${k}_{V}$ are the local-area modulus, global-area modulus, and global-volume modulus, respectively. As stated before, ${\overrightarrow{n}}_{ABC}$ is the unit vector normal to the triangle *ABC*^23^.

#### Viscous damping coefficient

Since the cell membrane has a lipid layer within its bilayer, it exhibits viscoelastic behaviour. In OIF the membrane viscosity was implemented using a Newtonian viscous damper^15^:

13 $${\overrightarrow{F}}_{visc}\left(A\right)=-{k}_{visc}\left({\overrightarrow{v}}_{A }.{\overrightarrow{p}}_{AB}\right){\overrightarrow{p}}_{AB}$$

where ${k}_{visc}$ is the viscous damping coefficient, and ${\overrightarrow{v}}_{A}$ is the velocity of node *A*.

#### Cell-wall interaction

At a constant pressure drop between the two ends of the constricted microchannel, the passage time (entry time + transit time) of a cell depends on cell deformability as well as cell-wall surface friction. Furthermore, in ESPResSo repulsive forces were defined between the nodes of the cell and microchannel wall to avoid cell penetration into the microchannel wall in order to model the behaviour of the cell near the walls as^15^:

14 $${\overrightarrow{F}}_{r}\left(d\right)=a\frac{n}{{d}^{(n+1)}}\overrightarrow{m}, d<{d}_{cut}$$

where $d$ is the distance between the nodes of cell and wall, ${d}_{cut}$ is the threshold of repulsive force activation, $a$ and $n$ are the repulsive force coefficients, and $\overrightarrow{m}$ is the unit vector pointing from the wall node to the cell node^15^. The repulsive-force parameters were considered to be constant in all simulations in this study.

On the other hand, since originally ESPResSo and OIF did not have friction-force implementation in the source code, to realistically model cell passing through a constriction, we defined the below friction force

15 $${\overrightarrow{F}}_{f}\left(A\right)={-\mu }_{f} a\frac{n}{{d}^{(n+1)}}\frac{{\overrightarrow{v}}_{A}}{\left|{\overrightarrow{v}}_{A}\right|}, d<{d}_{cut}$$

where ${\mu }_{f}$ is the surface friction coefficient.

### Numerical model of cell passage through a microfluidic constriction

In this study, our aim is to develop a validated spring-network model for cancer cells as well as a numerical tool that can accurately find the parameters of this model. The validated model with accurate parameters then can be used to model the deformation behaviour of cancer cells in various scenarios. We identified parameter values that enabled the model to replicate the experimental data^9^ of a cancer cell passing through a microfluidic constriction. In their study, a suspended microchannel resonator with a constriction was used to investigate the deformability and surface friction of various types of cancer cells including the human lung cancer cell line H1975. The constriction was 6 μm in width, 15 μm in depth, and 50 μm in length with a 45° tapered entrance. Cells were kept in cell solution (RPMI 1640) at 37 °C and sent through the microchannel with a constant pressure drop that drove the flow in the fluidic microchannel. The flow rate of the blank media at the drop pressure of 1.5 psi was reported to be 38 μL/h. The measurements of the passage of H1975 were performed at two different drop pressures of 1.8 psi and 0.9 psi^9^. The flow rates at these two drop pressures were calculated using Poiseuille’s law as listed in Table 2. In this table, the entry time is the required time for the cell to completely enter the constriction and the transit time is the time the cell takes to travel in the constriction and to completely exit it. Because their experimental results^9^ were reported based on the cell buoyant mass, to find the cell radius in their experimental data, we used the following equation that relates the cell radius $r$ to its buoyant mass:

16 $${m}_{b}=\frac{4\pi {r}^{3}}{3}(\rho -{\rho }_{f})$$

where, ${m}_{b}$ is the cell buoyant mass, $\rho$ is the cell density, and ${\rho }_{f}$ is fluid density^9^. We modelled the cancer cell as a sphere with the radius of 6.5 μm and discretized with a triangular surface mesh with 393 nodes. We performed Python scripting in ESPResSo to create the geometrical setup of the microchannel and the computational lattice for our simulations. Table 3 shows the parameters used in our numerical simulations of cancer cell motion and deformation in the microchannel.

**Table 2:** The extracted entry time and transit time for the cell radius of 6.5 μm at two flow rates.

Experiment number	Flow rate ($\frac{{\varvec{\mu}}{\varvec{L}}}{{\varvec{h}}}$)	Entry time (μs)	Transit time (μs)
1	22.8	17,540	3740
2	45.6	2470	526

**Table 3:** Parameters used for modelling cancer cell deformation and motion in the microchannel.

Parameters	Symbol	Value	Unit
Time step	$\Delta t$	0.01	$\mu s$
Lattice resolution	$\Delta x$	1	$\mu m$
RPMI 1640 kinetic viscosity	$\upsilon$	0.785	$mPa\cdot s$
RPMI 1640 density	${\rho }_{f}$	1006	$\frac{kg}{{m}^{3}}$
Reference friction	${\xi }_{ref}$	0.922	$\left(nN\cdot \frac{s}{m}\right)$
Cancer cell density	$\rho$	1050	$\frac{kg}{{m}^{3}}$
Repulsive force activation threshold	${d}_{cut}$	0.1	$\mu m$
Repulsive force scale coefficient	$a$	0.0001	-
Repulsive force coefficient	$n$	1.2	-

Because, in the experiments, walls of the microfluidic channels were not lined with endothelial cells, receptor-ligand interactions between the CTCs and walls did not exist and therefore they were not considered in the present model.

**Figure 1:**
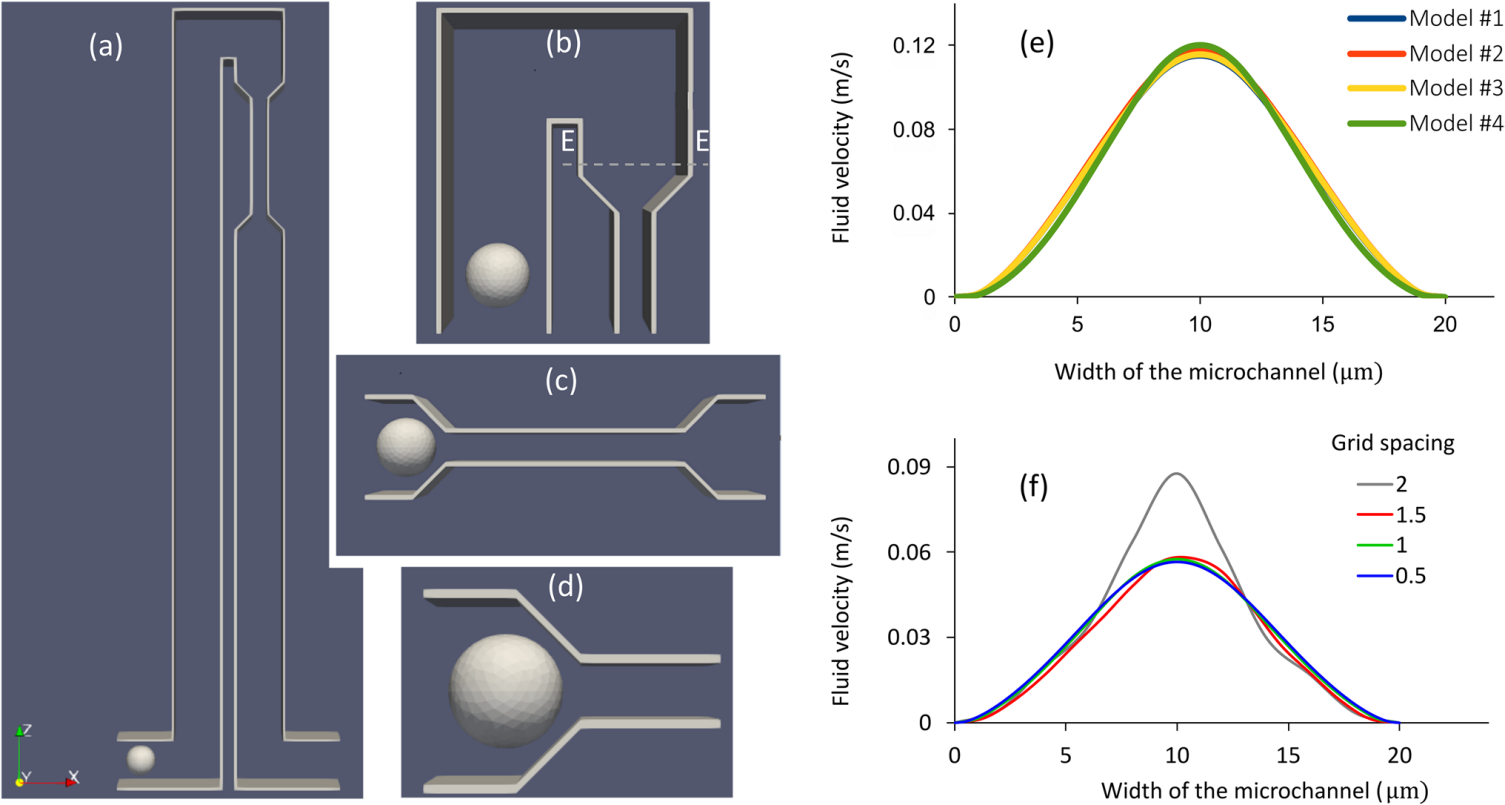
Investigations for selecting a computationally efficient geometrical domain to simulate the passage of cancer cells through a microchannel. The selected geometrical domain would be used in the computationally expensive parameter-identification process. Four different models were investigated: (**a**) Model #1: the entire microchannel setup used in experiments of Byun et al.^9^, (**b**) Model #2: part of the entire microchannel setup of Model #1, (**c**) Model #3: the entire constriction part of Model #1, (**d**) Model #4: the entry part of the constriction of Model #1, (**e**) Comparison of fluid velocities at the depth of 7 µm of the microchannel at cross-section E–E in (**b**) for the four models, and (**f**) The fluid velocity with different lattice grid spacing sizes for the flow rate of 22.8 µL/h at the cross section E–E shown in panel (**b**). Fluid flow is not sensitive to the grid spacing size for the grid spacing of 1.5 µm and finer.

To achieve the goal of this study, a large number of numerical simulations should be executed repeatedly to find accurate model parameters. Since numerical simulation of the whole microchannel setup was computationally expensive, performing the calculations with a smaller model with an acceptable computational accuracy could greatly speed up the parameter identification process. We performed numerical simulations of the motion and deformation of lung cancer cells in four geometrical domains shown in Fig. 1. In this figure, Model #1 (panel a) includes the entire fluid passage, while Models #2, #3 and #4 (panels b, c and d) only include a portion of Model #1.

Figure 1e shows the fluid velocity profiles at the depth of 7 μm at the cross-section E-E (shown with a dashed line in Fig. 1b) for the flow rate of 45.6 µL/h. All three partial models, that have smaller geometrical domain compared to the complete microchannel model (Model#1), have velocity profiles very close to that of Model#1. The numerical results of lung cancer cell entry time along with the required simulation runtime were compared in the four models with similar viscoelastic parameters for four various combinations of the parameter values shown in Table 4. The calculated cell entry time and simulation runtime of Models #2, 3 and 4 against those of Model #1 were compared in Table 4. For Models #2, 3 and 4, the average errors were 4%, 4.2% and 1419.2%, respectively, while the average runtimes were 1509.25 s, 267.25 s, and 2161.5 s, respectively. Because of its substantial error, Model #4 cannot be reliably used instead of Model#1. Although Model #2 was 0.2% more accurate than Model #3, its runtime was over five times of that of Model #3. Since Model #3 can sufficiently and efficiently represent Model #1, we used Model #3 in the calculations of the present study.

**Table 4:** Comparison of the four models in Fig. **1** in terms of entry time and run time for four combinations of viscoelastic parameters.

Viscoelastic Coefficients						Model #1		Model #2		Model #3		Model #4	
${k}_{s}$	${k}_{b}$	${k}_{al}$	${k}_{ag}$	${k}_{V}$	${k}_{visc}$	Entry time (s)	Run time (s)	Entry time (s)	Run time (s)	Entry time (s)	Run time (s)	Entry time (s)	Run time (s)
0.5	0.8	0.05	0.9	0.9	1.5	245	40,565	245	1326	250	182	310	103
1.3	1.6	0.5	1.0	1.5	2.0	410	46,346	390	1567	430	271	1390	409
1.3	1.6	1.0	1.0	2.0	4.0	450	47,747	435	1536	470	292	4250	1253
1.0	1.6	1.0	2.0	2.0	4.0	500	49,498	520	1608	530	324	23,340	6881

Figure 1f illustrates the fluid velocity profile at the E–E cross section (Fig. 1b) for the flow rate of 22.8 µL/h with different lattice grid spacing for the fluid domain. As Fig. 1f shows the fluid flow is not sensitive to the grid spacing of equal or less than 1.5 μm. Therefore, in this study the grid spacing of 1 μm was used for the fluid domain to have a high accuracy while keeping the computational cost manageable.

The deformation of the spring-network cell model, however, is sensitive to the mesh size so that all parameters should be changed when the mesh size is changed. Therefore, for each mesh size of the cell, the proposed parameter identification process should be repeated.

### Genetic algorithm

The spring network model for the cell includes several parameters which need to be set accurately and because the parameters are dependent on one another, finding the best set of parameters to imitate large deformations of cells is a complicated task. In this work, we propose an optimization-based method to accurately identify the parameters of spring-network models of cells. We defined an error function and developed an optimization algorithm based on GA to accurately and automatically identify parameters to minimize the error of the model results compared to the experimental results.

The steps of the GA code are as follows:

1. Random n = 128 bits fractional binary numbers were generated for each model parameter to constitute the initial population for each iteration. The fractional binary numbers were converted to fractional decimal numbers using the following equations:
  17 $${(0.{a}_{0}{a}_{1}\dots {a}_{n-1})}_{2}={({a}_{0}\times {2}^{-1}+{a}_{1}\times {2}^{-2}+\dots +{a}_{n-1}\times {2}^{-n})}_{10}= {q}_{10}$$
  18 $$K={K}_{L}+\left({K}_{U}-{K}_{L}\right)\times {(q}_{10})$$
  where ${K}_{L}$, ${K}_{U}$ are the lower bound and the upper bound given in Table 5. As an example, the following binary fraction for ${K}_{s}$ with ${K}_{L}=0.0001$ and ${K}_{U}=10$ and n = 128, 0.01111111110110101010100010001101111001001111001000111011011110110101011010101010000100001101010110110110101001001111000000110110 is the decimal ${K}_{s}$ of 4.994. The initial population includes 40 different chromosomes for each iteration.
2. Parents were selected from the initial population and then crossover were performed for probabilities of higher than 0.8. Mutations were performed for each bit for probabilities of higher than 0.8.
3. Binary numbers were converted to decimal numbers according to the upper and lower bounds of each parameter as provided in Table 5.
4. Numerical simulation of the corresponding experiment was conducted for each set of parameters, and then the outputs were stored and compared with the experimental results.
5. Each iteration had about 400 distinct parameter sets. In order to generate random numbers based on the best results of the previous iteration, 20 best sets of parameters of the previous iteration, were chosen based on the error function. They were stored and added to the initial population of the current iteration.
6. The algorithm stopped if one of the below conditions occurred:
  - The error function value is less than a user-defined threshold.
  - The best result remains unchanged for 20 successive iterations.

**Table 5:** Upper and lower bounds for model parameters.

	${k}_{s}$	${k}_{b}$	${k}_{al}$	${k}_{ag}$	${k}_{V}$	${k}_{visc}$	${\mu }_{f}$
Lower bound	0.0001	0.0001	0.0001	0.1	0.1	0.1	0.0001
Upper bound	10.0	10.0	10.0	10.0	10.0	10.0	1.0

The parameter identification process and the required numerical simulations were performed on the Graham supercomputer of Compute Canada. Up to 300 intel Xeon E5-2683V4 cores with the base frequency of 2.1 GHz were used in parallel. Each of the single simulation of 1 ms (10^5^ time steps) of the cell motion on the Graham supercomputer when allocating one core and 1024 MB RAM took around 1850s.

### Data visualisation and post processing

For visualising the microfluidics walls, cancer cell and fluid flow, coordinates and fluid velocity were extracted from the OIF module in .vtk files. These files were then visualised using ParaView open-source software (version 5.8). Other cell-related information such as cell length, cell area strain and cell velocity were calculated by defining appropriate filters in ParaView. No smoothing or averaging was used in presenting the data.

## Results

### Validation of the parameter identification method for spring-network model of cells

**Figure 2:**
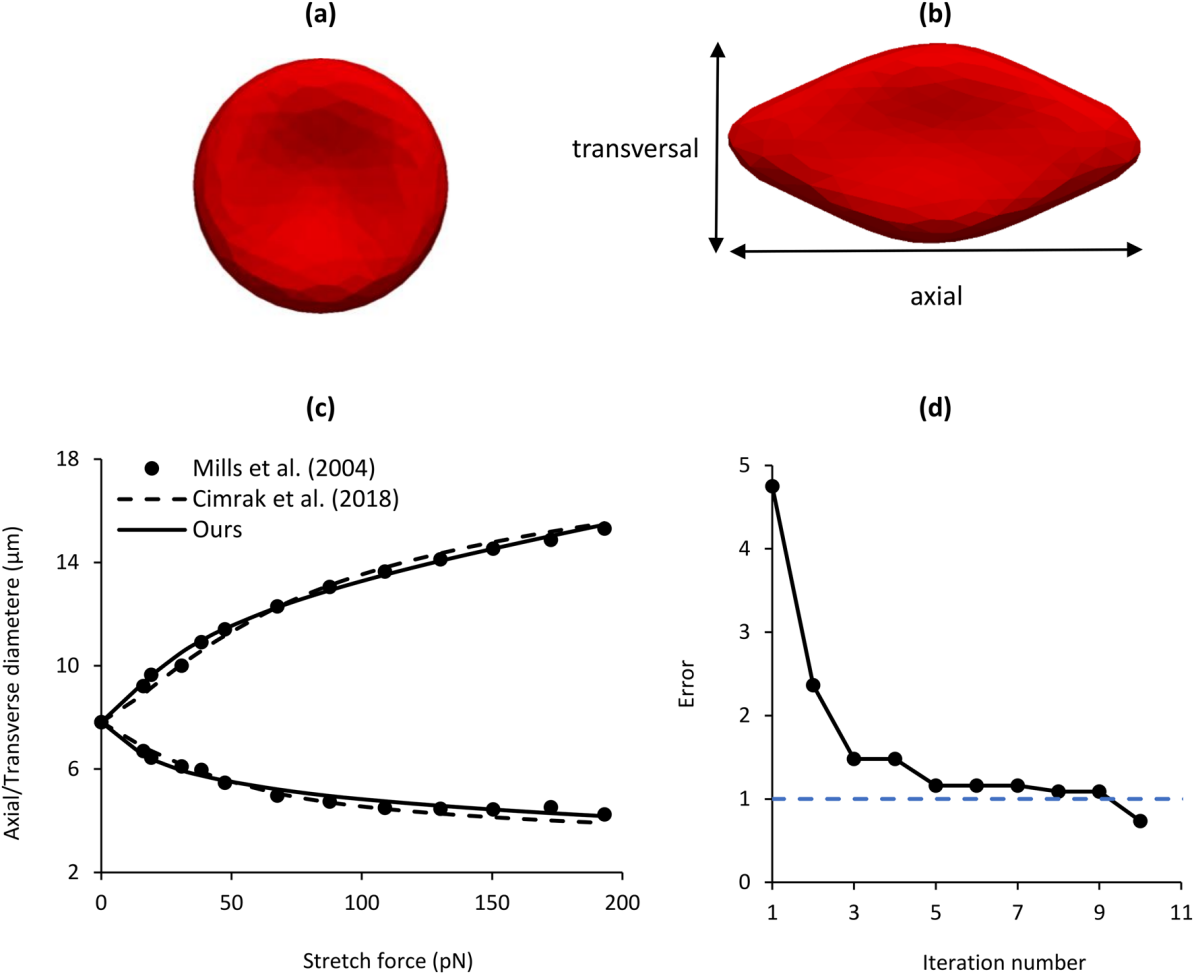
Validation of the proposed parameter identification method using stretch experiments of the red blood cell (RBC) with 374 nodes (**a**) relaxed state, (**b**) stretched state, (**c**) comparison of RBC stretch results obtained (1) experimentally by Mills et al.^3^ using optical tweezers, (2) numerically by Cimrak et al.^15^ using parameters given in Table 6 and (3) numerically using the parameters identified using the proposed genetic algorithm identification method. The model with parameters identified using the proposed method can replicate experimental data more closely than the model by Cimrak et al. (**d**) error minimization evolution of the proposed method. The main drop in the error occurred in the first five iterations and the five next iterations fine tuned the parameters and decreased the error to less than the threshold of one.

In order to assess the sufficiency of our proposed GA-based method for identifying the values of parameters of the spring-network model of cells, we used the method to identify the parameters of the RBC. We used the RBC stretch experiment performed using optical tweezers by Mills et al.^3^. Figure 2a shows the 3-D model of the RBC with 374 mesh nodes that we used in our calculations. The RBC was stretched in the axial direction as shown in Fig. 2b. We used the following error function

21 $$Error=\sum_{i=1}^{12}\left({\left({s}_{i}^{a}-{e}_{i}^{a}\right)}^{2}+{\left({s}_{i}^{t}-{e}_{i}^{t}\right)}^{2}\right)$$

where ${s}^{a}$ and ${s}^{t}$ are the axial and transversal diameter of RBC, respectively, which were calculated numerically at the last time step. Also, ${e}^{a}$ and ${e}^{t}$ are the axial and transversal diameter of the RBC measured in stretch experiment with optical tweezers^3^. The stop condition error threshold was defined to be one^15^.

Figure 2c compares the experimental results of Mills et al.^3^ with the numerical result of our stretching simulation using the parameter set that our proposed method identified (Table 6) by minimizing the error function of Eq. (16). As Fig. 2d shows, the error function greatly decreased from 4.8 to close to 1 after only five iterations and then decreased to below the threshold of one after ten iterations and the calculations stopped and model parameters were identified (Table 6). Figure 2c also shows the numerical result that Cimrak et al.^15^ obtained using their manually adjusted parameters given in Table 6 on an RBC with the same number of nodes and mesh configuration as used in our simulation and shown in Fig. 2a. As Fig. 2c and Table 6 show, compared to the parameters obtained by Cimrak et al.^15^, the parameters identified by our GA method enabled closer replication of the experimental results with a substantially lower error (0.73 versus 1.46).

**Table 6:** Parameter values for healthy RBC identified using the stretch experimental data of Mills et al.^3^.

	${k}_{s}$	${k}_{b}$	${k}_{al}$	${k}_{ag}$	${k}_{V}$	Error
Cimrak et al. (2018)	0.006	0.008	0.001	0.5	0.9	1.46
Proposed GA method	0.0016	0.0569	0.0011	0.281	2.94	0.73

### Parameter identification of the spring-network model of cancer cell

In this section, we used our proposed GA method to identify parameters of the human lung cancer cell (H1975). While the time the cell takes to squeeze and completely enter the constriction (entry time) is highly dominated by the deformability characteristics of the cell, the time the cell takes to travel inside the constriction (transit time) is affected by both surface friction and the deformability of the cell^9^. We, therefore, ignored the surface friction effects in calculating the entry time and first identified the viscoelastic parameters during the entry phase by minimizing the following error function:

22 $${Error}_{1}=\sum_{n=1}^{{n}_{t}}\left|1-{\left(\frac{{ET}^{s}}{{ET}^{e}}\right)}_{n}\right|$$

where ${ET}^{s}$, and ${ET}^{e}$ are numerically calculated entry time, and experimentally measured entry time, respectively. Here, ${n}_{t}$ is equal to 2 for using the entry time at two flow rates.

As described in “Spring-network model of cell”, the surface friction force is activated when the distance between the wall and cell nodes is less than ${d}_{cut}$. We observed that the number of cell nodes which have the active surface friction is not the same in different flow rates. Hence, the surface friction coefficient was identified for each flow rate separately according to the following error function:

23 $${Error}_{2}^{i}=\left|1-\frac{{TT}_{i}^{s}}{{TT}_{i}^{e}}\right| \, for \, i=1, 2$$

where $i$ is the experiment number according to Table 2 and ${TT}^{s}$, and ${TT}^{e}$ are numerical transit time and experimental transit time, respectively.

Table 7 shows the identified values for the friction coefficients of the lung cancer cell with the radius of 6.5 μm discretized with 393 nodes using experimental results at two different flow rates (given in Table 2).

**Table 7:** The best values for the spring-network model parameters of the lung cancer cell.

${k}_{s}$	${k}_{b}$	${k}_{al}$	${k}_{ag}$	${k}_{V}$	${k}_{Visc}$	${\mu }_{f}^{1}$	${\mu }_{f}^{2}$
0.073	1.649	0.969	3.084	0.823	2.481	0.0118	0.0009

Table 8 shows the entry time, transit time and the calculated errors at these two flow rates.

**Table 8:** Errors and calculated entry and transit times for the best values of the model parameters for the lung cancer cell at the two flow rates of Table 2.

$\mathrm{i}$	Calculated entry time (μs)	Calculated transit time (μs)	${Error}_{1}$	${Error}_{2}^{1}$	${Error}_{2}^{2}$
1	16,595	3497	0.206	0.069	0.011
2	2094	532			

### Steps of cancer cell deformation in constriction

The model of the cancer cell deformation rigorously validated against the experimental data, presented in “Parameter identification of the spring-network model of cancer cell”, enables us to study details of the deformation process inside the constriction. The model can provide information about the deformation of cancer cells in the constriction at the length scale and time scale that are not accessible in the experimental measurements. Understanding the deformation steps, that the cancer cell undergoes, is key for our study in the next sections and for developing future microfluidic devices for cancer cell characterization and diagnosis.

**Figure 3:**
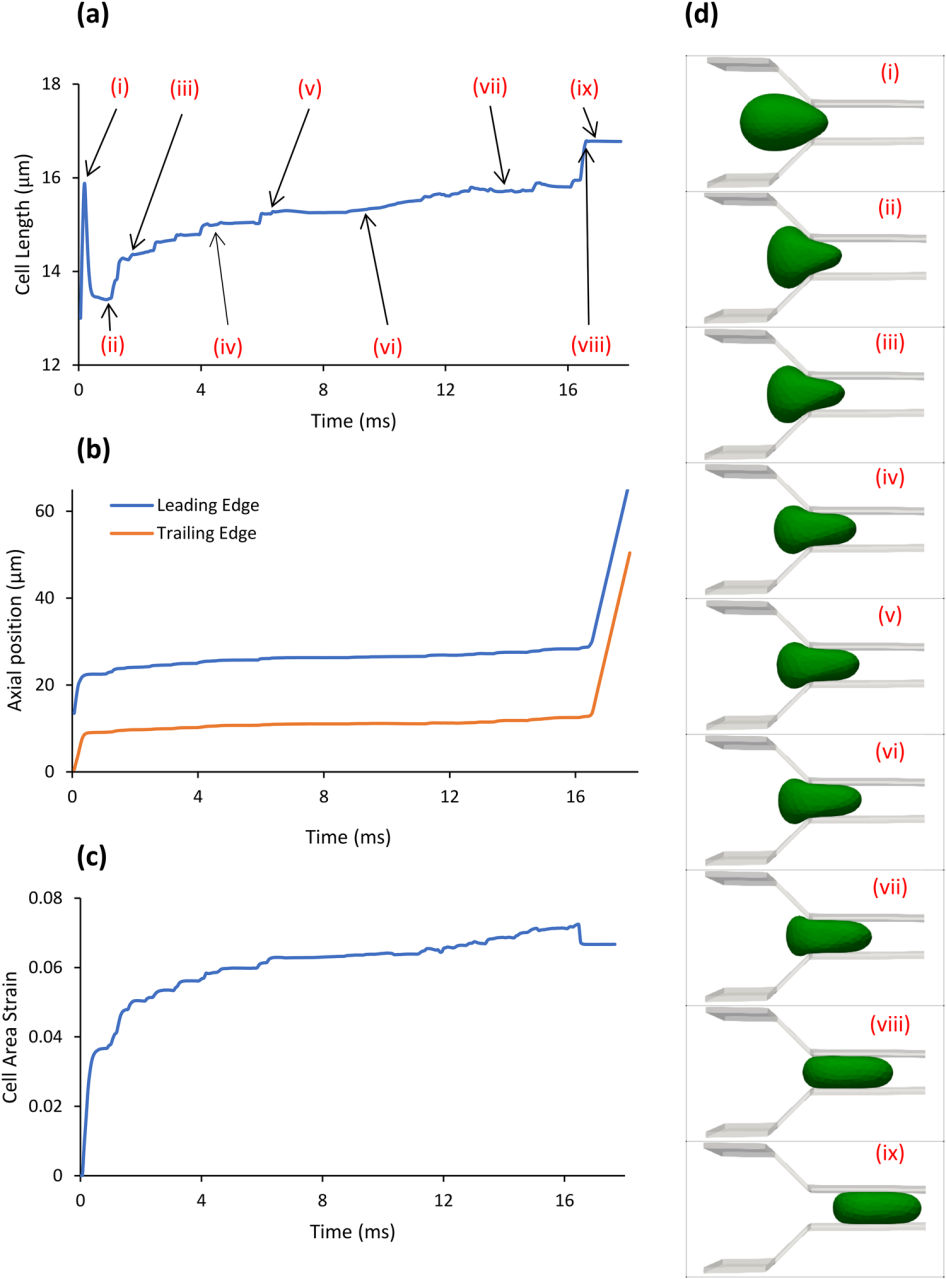
Deformation and motion of a lung cancer (H1975) in a microfluidic channel with flow rate of 22.8 µL/h calculated using the validated model. (**a**) evolution of the cell length as the cancer cell advanced in the microchannel, (**b**) motion of the cell in the microchannel represented by the trajectories of its leading and trailing edges, (**c**) evolution of the area strain of the cell (change in the cell area relative to its undeformed shape divided by its undeformed initial area) as the cell advanced in the microchannel, and (**d**) snapshots of the 3-D model of cell progression in the microchannel at nine instances. The instances shown in (**d**) are indicated by arrows in the evolution of cell length in panel (**a**). The cell underwent a rather sudden elongation in Step i that was accompanied by a jump in the area strain as well. The cell length then quickly decreased upon start of squeezing of the cell to the constriction between Steps i and ii but the area strain continued to grow as the cell expanded in the direction normal to the view provided in panel (**d**). Between Steps ii and viii, the cell continued to deform as it is evident from panels (**a**,**c**,**d**) while it stayed almost at the same position as panel (**b**) shows. After complete entry of the cell in the constriction, the cell transited in the constriction in Step ix that was much shorter than the entry time as the sloped trajectory lines of panel (**b**) near the end of the time show.

Figure 3 shows the progression of the deformation of the lung cancer cell in the constriction in terms of cell length (panel a), position of the cell indicated by its leading and trailing edges (panel b), cell area strain (panel c), and the 3-D view of the cell at nine key instances of passage (panel d). The area strain was calculated as the difference of the instantaneous area with the resting area divided by the resting area. In this figure, the flow rate of 22.8 $\mu L/h$ drove the cell. At the onset of entry (Step i), a sudden 22% elongation (Fig. 3a) and a sudden increase of 0.036 in the area strain (Fig. 3c) occurred. The sharp slopes in the leading and trailing edge traces (Fig. 3b) show that the cell moved inside the channel while deforming from a sphere to an ovoid (Fig. 3d).

Between Steps i and viii, the gentle slopes of the leading and trailing edge traces (Fig. 3b) show continuous but sluggish progression of the cell into the constriction. However, details of the deformation are not visible in the leading and trailing edge traces. Pushing the cell against the constriction wall between Steps i and ii (Fig. **3**d), caused the cell length to drop to near only 3% longer than the original diameter (Fig. **3**b) but the almost constant area strain shows that the cell expanded in the direction normal to the view of Fig. **3**d. Between Steps ii and iii, the cell underwent another rapid elongation (7% increase) and a rapid increase in the area strain (to 0.05) as Fig. **3**a,c show. The major portion of the time (89%) during entry was spent between Steps iii and viii when the cell underwent small gradual elongation and gradual increase in the area. In Step vi, the area strain stayed almost constant while the length gradually increased. In Step vii, the cell ceased the constant area regime and eventually completely entered the constriction in Step viii. This was accompanied by a sudden 0.003 decrease in the area strain while the cell suddenly elongated in the narrow constriction to the eventual 29% increase compared to the original diameter. Step ix is the transit step during which the cell length and area strain remained almost constant while the cell rapidly travelled the narrow constriction as demonstrated by the steep traces of the leading and trailing edges in Fig. **3**b.

**Figure 4:**
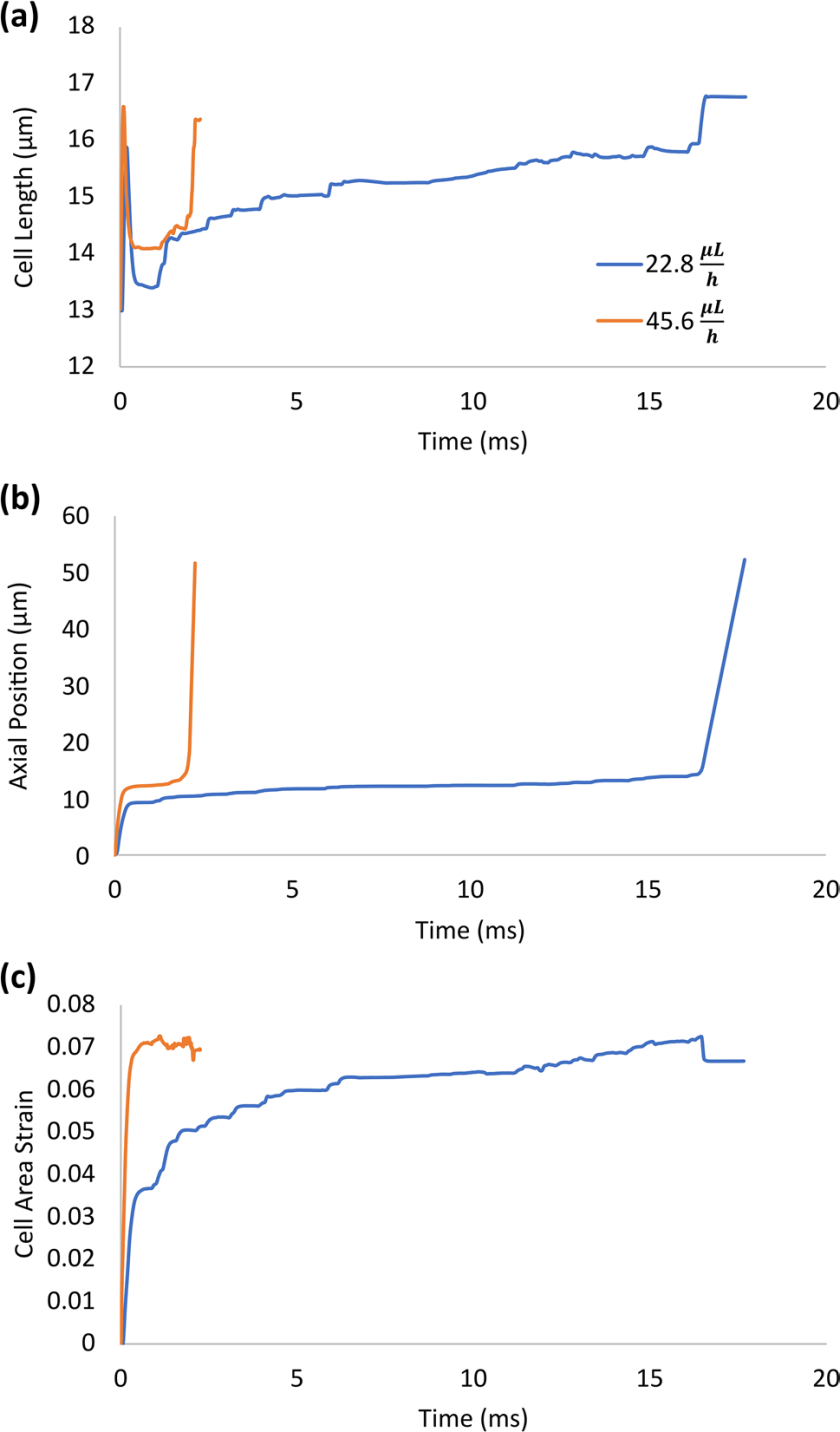
Comparison of deformation and motion of a lung cancer (H1975) in a microfluidic channel at two flow rates of 22.8  and 45.6 µL/h. (**a**) evolution of the cell length as the cancer cell advanced in the microchannel, (**b**) motion of the cell in the microchannel represented by the trajectories of its centre of mass, and (**c**) evolution of the area strain of the cell (change in the cell area relative to its undeformed shape divided by its undeformed initial area) as the cell advanced in the microchannel. The evolution of the cell at the low flow rate and the steps are marked in Fig. 3 and here it is shown for reference. The cell underwent a higher initial elongation and area strain in Step i with the high flow rate than it did with the low flow rate. The initial high area-strain value, that the cell reached to in Step i with the high flow rate, stayed almost constant during the entry steps. However, with the low flow rate, the area strain gradually increased during the entry steps until it eventually reached to the high value that the cell reached to Step i with the high flow rate.

At the higher flow rate of 45.6 $\mu L/h$, the steps described above are completed faster as panels of Fig. **4** show (steps are labeled in Fig. **3**). The initial stretch of the cell in Step i was more at the higher flow rate than it was at the low flow rate of 22.8 $\mu L/h$ as Fig. **4**a shows (16.6 vs 15.9 µm). Furthermore, the area strain in Fig. **4**c shows a higher jump of 0.07 in Step i than the jump of 0.05 that the cell underwent at the lower flow rate. Between Steps i and viii, unlike at the lower flow rate that the area strain showed the overall continued growth 0.05 to about 0.07, at the high flow rate, the area strain remained almost constant near 0.07 with small undulations (Fig. **4**c). Figure **4**b shows the axial location of the centre of the cell at both flow rates. As this figure shows, between Steps i and ii, the cell entered further into the narrow constriction at the high flow rate than it did at the low flow rate. This is evident from the cell-centre axial locations of 11.6 µm with the high flow rate versus 8.2 µm with the low flow rate (both at 0.3 ms). This observation is consistent with the cell length plots of Fig. **4**a where between Steps i and ii, the cell always elongated more with the high flow rate than it did with the low flow rate. As Fig. **4**a shows, while the time span between Steps i and ii at the two flow rates were almost the same, cell deformation evolution between Steps ii and viii were compressed in time at the high flow rate compared to that at the low flow rate. The straight horizontal lines (Step ix) at the end of the plots in Fig. **4**a,c show that the transit time was much shorter at the high flow rate than it was at the low flow rate. This is consistent with the almost vertical line at the end of the cell-centre trace of Fig. **4**b that indicates very short transit time compared with the steep-sloped end of the cell-centre trace at the low flow rate.

### Parsimony of spring-network model for cancer cells

While the elastic parameters used in the model that we present here for cancer cells are widely used in spring-network models of cells, the viscosity is not included in some cell models^24^. Also, the friction between the cell and the wall is usually ignored in models^9,14^. In the following sections, we investigated how ignoring these two parameters, in order to reduce the number of the parameters of the cancer-cell model, can affect the validity of the model results.

#### Effects of membrane viscosity on cell deformation in constriction

Considering viscosity of the cell membrane is crucial in developing a sufficient model of cancer cell motion and deformation. Indeed, the cell deformation in the constrictions showed flow-rate-dependent sensitivity to membrane viscosity as demonstrated in this section.

**Figure 5:**
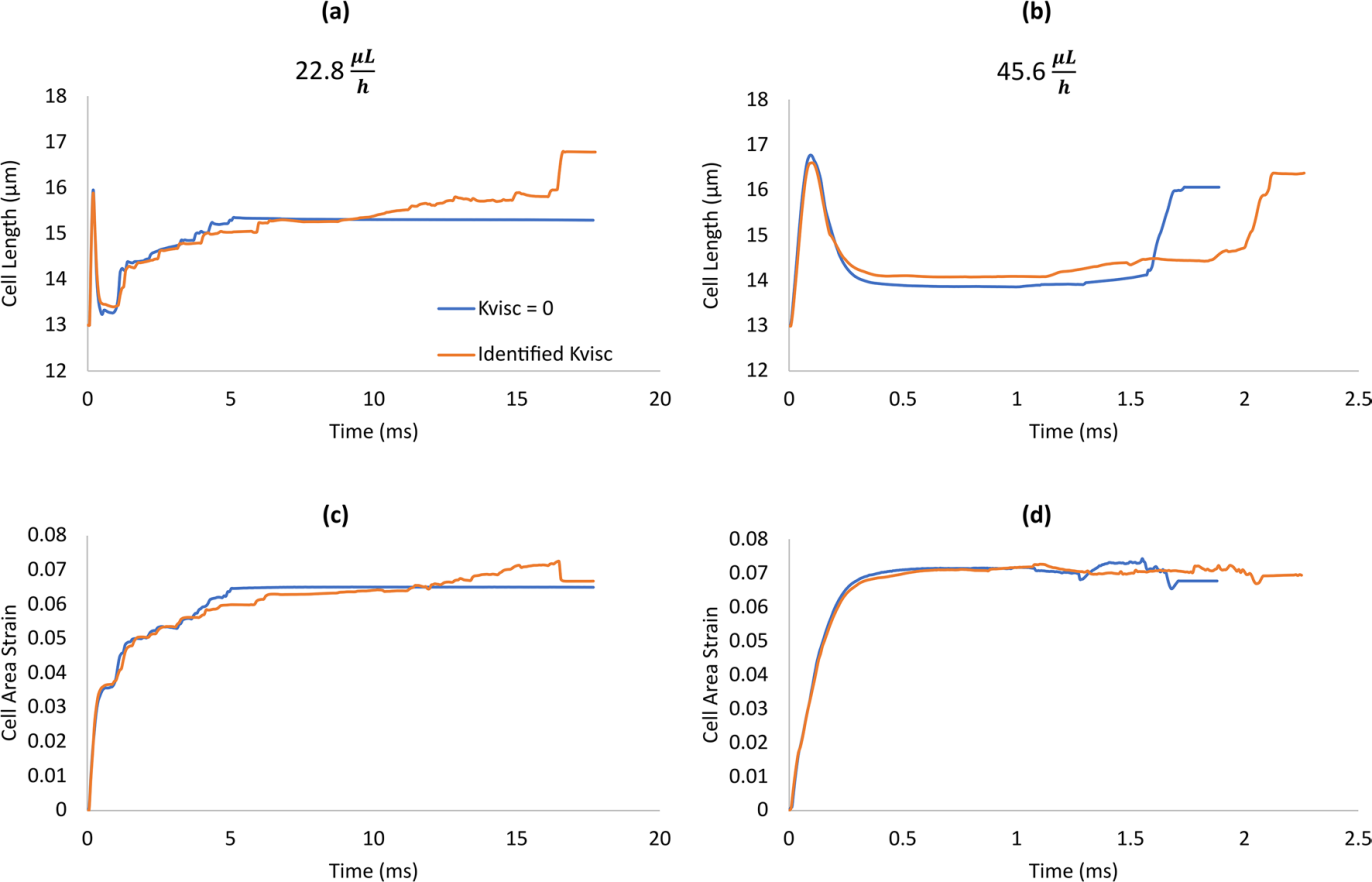
Effects of membrane viscosity on cancer-cell deformation in constriction at two flow rates. (**a**,**c**) evolutions of the cell length and area strain at 22.8 µL/h, (**b**,**d**) evolutions of the cell length and area strain at 45.6 µL/h. The area strain is defined as the change in the cell area relative to its undeformed shape divided by its undeformed initial area. Membrane viscosity had flow-rate dependent effects of the motion and deformation of the cell. Neglecting the viscosity caused overestimation and underestimation of the entry time to the constriction at the low and high flow rates, respectively.

**Figure 6:**
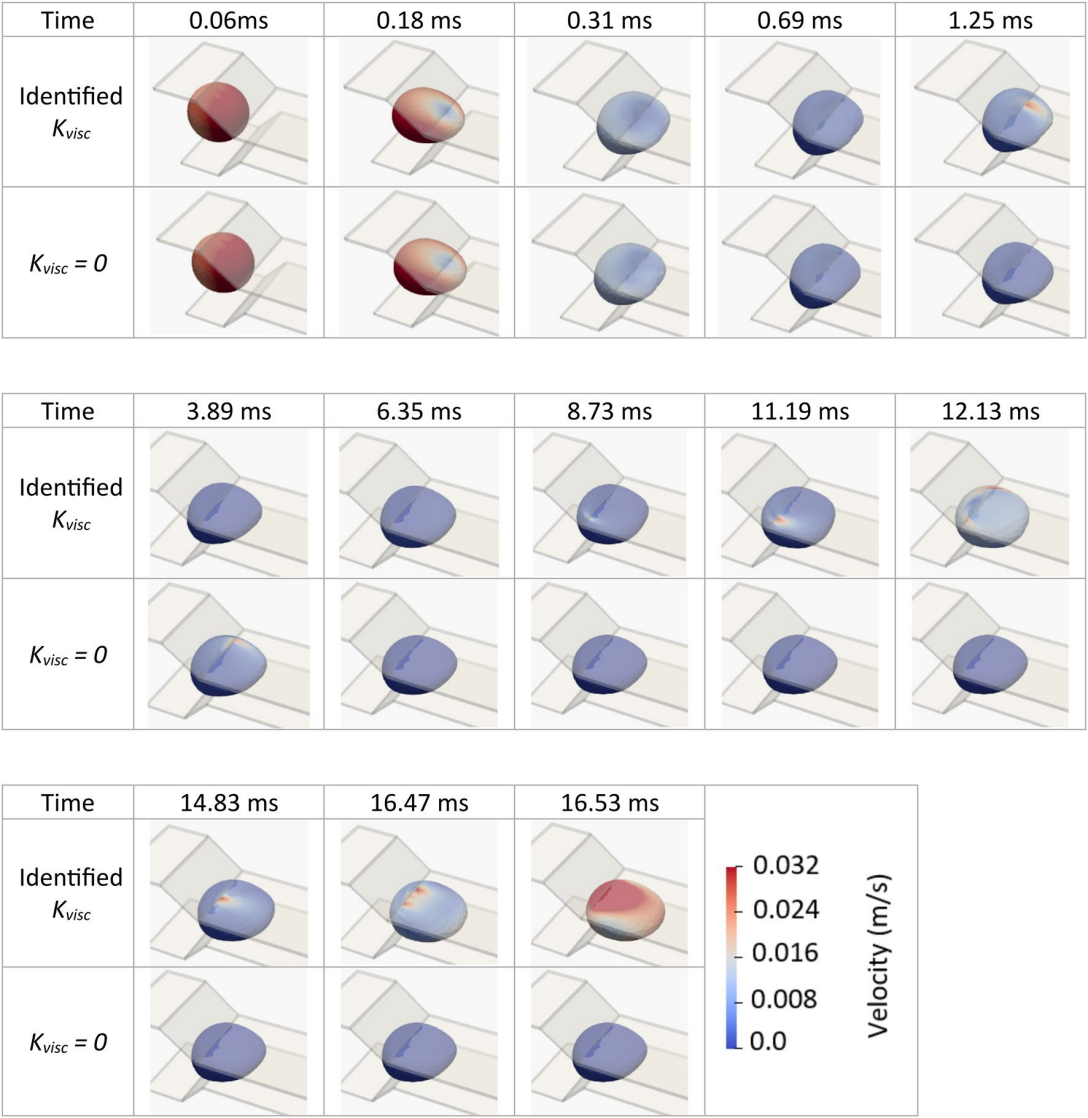
Effects of the viscosity of cell membrane on the 3-D motion and deformation of a lung cancer cell in a microfluidic constriction at the flow rate of at 22.8 µL/h. Neglecting the viscosity caused the cell to get stuck at the constriction entrance after the 6.53 ms while the cell with correctly identified viscosity underwent drastic deformations until it successfully entered the constriction at 16.53 ms.

At the low flow rate (22.8 *µL/h*), ignoring the cell membrane viscosity reduced the deformation and changed the timing of deformation. Both the cell length (Fig. **5**a) and area strain (Fig. **5**b) show, although the cell without viscosity reached to Step v (defined in “Steps of cancer cell deformation in constriction”) sooner than the cell with the correct viscosity did (5.2 vs 6.2 ms respectively). However, it remained in Step vi (indicated with the straight horizontal line after 5.2 ms) while the cell with the correct viscosity finished all steps and exited the microchannel. Consistently, the 3-D views of the cell in Fig. **6** show that the cell without viscosity remained unchanged at the entrance of the narrow constriction at the shown instances of 6.53 ms and beyond. Meanwhile, the cell with the correct viscosity exhibited a series of deformations that eventually resulted in the entrance to the narrow constriction at 16.53 ms.

On the other hand, at the high flow rate (45.6 *µL/h*), the cell with ignored viscosity reached Step ii sooner than the cell with the correct viscosity did (1.6 vs 2 ms) as it is clear in the cell length plot of Fig. **5**c and the area strain plot of Fig. **5**d. The cell with ignored viscosity completed all subsequent steps sooner than the cell with the correct viscosity did and exited the microchannel with the passage time of 1.9 ms compared to that of 2.2 ms for the cell with correct viscosity.

#### Effects of friction on cell passage in constriction

**Figure 7:**
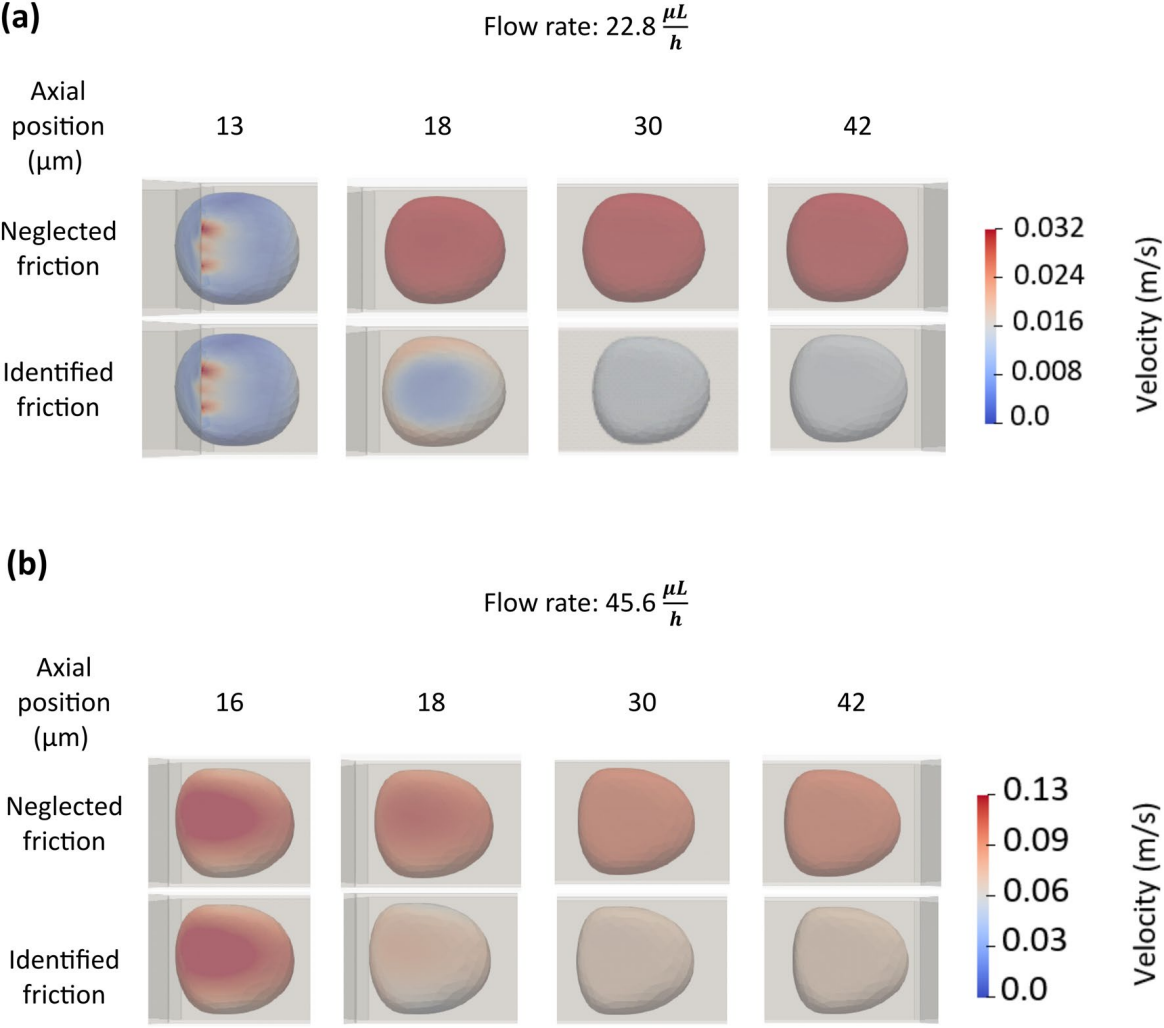
Effects of friction between the walls of the microchannel and the cancer cell on the cell motion and deformation during its transit in the constriction (Step ix in Fig. **3**) of motion at the flow rates of 22.8 µL/h (**a**) and 45.6 µL/h (**b**). The cell is viewed from the top (perpendicular to the microchannel axis). Because neglecting friction caused the cell to pass very fast, instead of time-matched states, axial location-matched states are shown. At both flow rates, the cell velocity magnitude at any location in the constriction was always overestimated when the friction was ignored.

In this section, we demonstrate that in order to develop accurate spring-network models of cancer cells in interaction with a wall, using an accurate friction coefficient is crucial and this parameter should not be ignored. Figure **7** shows the transit step (Step ix as defined in Fig. **3**) for the flow rates of 22.8 and 45.6 $\mu L/h$ at four axial locations along the narrow constriction. In both Fig. **7**a,b, the top rows show the cell motion states simulated while ignoring friction and the bottom rows show the cell motion states simulated using the friction coefficient identified for the flow rate (Table 7). We present the location-matched states of cells because the cell in the frictionless condition travelled so fast that time-matched comparison is not useful. The cell is coloured with the velocity magnitude.

At the low flow rate (22.8 $\mu L/h$), at the 18 µm axial location, the cell with identified friction still had nonuniform velocity with the maximum speed of about 2 cm/s while the frictionless cell already exhibited rigid body motion with the uniform speed of 3 cm/s. At this flow rate, both with-friction and frictionless cancer cells showed rigid-body motion at the 30 and 42 µm axial locations and the frictionless cell travelled twice as fast as the cell with correct friction did (at the constant speed of about 3 vs 1.5 cm/s). At the higher flow rate (45.6 $\mu L/h$), at the 18 µm axial location, cells in both conditions reached to rigid body and constant-speed motion. The frictionless and with-friction cells travelled at 10 and 7 cm/s, respectively. Ignoring the cell-wall friction caused a remarkable error in the transition time (81% and 55% error for 22.8 and 45.6 $\mu L/h$, respectively).

As explained in “Parameter identification of the spring-network model of cancer cell”, in the proposed parameter identification method, the friction coefficient should be identified separately for each flow rate. When instead of the friction coefficients individually identified for each flow rate, the average of the friction coefficients identified for each flow rate was used, the following was observed. For the higher flow rate simulation became unstable and crashed, since forces exerted on the cell membrane nodes were high at this flow rate. For the lower flow rate, using the average friction coefficient caused the calculated transit time to have %47 error in comparison with the experimental data.

## Discussion

Spring-networks have been increasingly employed for modeling the motion and deformation of red blood cells^24,27,37^, leukocytes^38^, platelets^39^ and cancer cells^40^. Spring-network models were already used for designing microfluidic chips^41^ and for such designs having a validated model with accurate parameters, as our method provides, is crucial. Furthermore spring-network models can be used to obtain fundamental understandings about metastatic spreading in the body and it is crucially important to use accurate parameters in these models. To the best of our knowledge, our proposed method is the first systematic method to find parameters of spring-network models to develop validated cancer cell models.

The parameters of the spring-network model of the RBC were determined by linking a continuum-mechanics model and a spring-network model of the cell^15^. However, that approach needs special assumptions which restrict the model from being general since the derivation is only valid for one specific geometry and cell type. Also, the parameters determined employing that approach do not accurately model the cell behaviour in comparison with experimental results and further calibration of the model parameters is required^37^. Our proposed method directly identifies the spring-network model parameters and does not rely on any other intermediate models or other restrictive assumptions.

To assess the effectiveness of the proposed method, we applied it to find parameters of the spring-network model of the RBC to imitate its deformation in the stretch experiments performed using optical tweezers. We showed that the parameters obtained using the proposed method enable closer replication of the experimental data compared to the reported parameters in the literature that were adjusted manually.

Ye et al.^42^ proposed functions that associated the transit time with bending modulus alone or shear modulus alone while treating all other parameters fixed. They later expanded the function to include both bending and shear moduli^41^. They studied effects of these two moduli on the entry time and fitted functions to relate the two moduli with transit time. Our approach in this work is different as we proposed a method to identify model parameters based on experimental data of cell motion in a microfluidic constriction.

The model of the cancer cell used in this study only includes the cell membrane represented by a network of springs. However, the deformation of the cell is also affected by the elasticity of the cell nucleus and cytoplasm. We identified the parameter values of the spring-network model of the cell using experimental data of the deformation of the entire cell. Therefore, the elasticity of the cell nucleus, cytoplasm and membrane are all lumped together and are captured by the parameters of the spring network of the cell.

For the two flow rates for which we had experimental data, the discrete friction coefficient was found to strongly depend on the flow rate. To correctly capture the physics of the interaction of the cell with the channel wall, in terms of the cell transit time, the friction coefficient was found to decrease from 0.0118 at 22.8 µL/h to 0.0009 at 45.6 µL/h. We observed that, compared to the low flow rate, at the high flow rate the cell nodes got closer to the channel wall causing a higher nodal normal repulsive force. The proposed algorithm balanced the intense normal repulsive force with a reduction in the coefficient of friction at the high flow rate (Eq. 15). These observations, however, were based on the experimental data at only two flow rates and should be verified by collecting experimental data at multiple flow rates and performing the parameter identification using the method proposed in this work.

**Figure 8:**
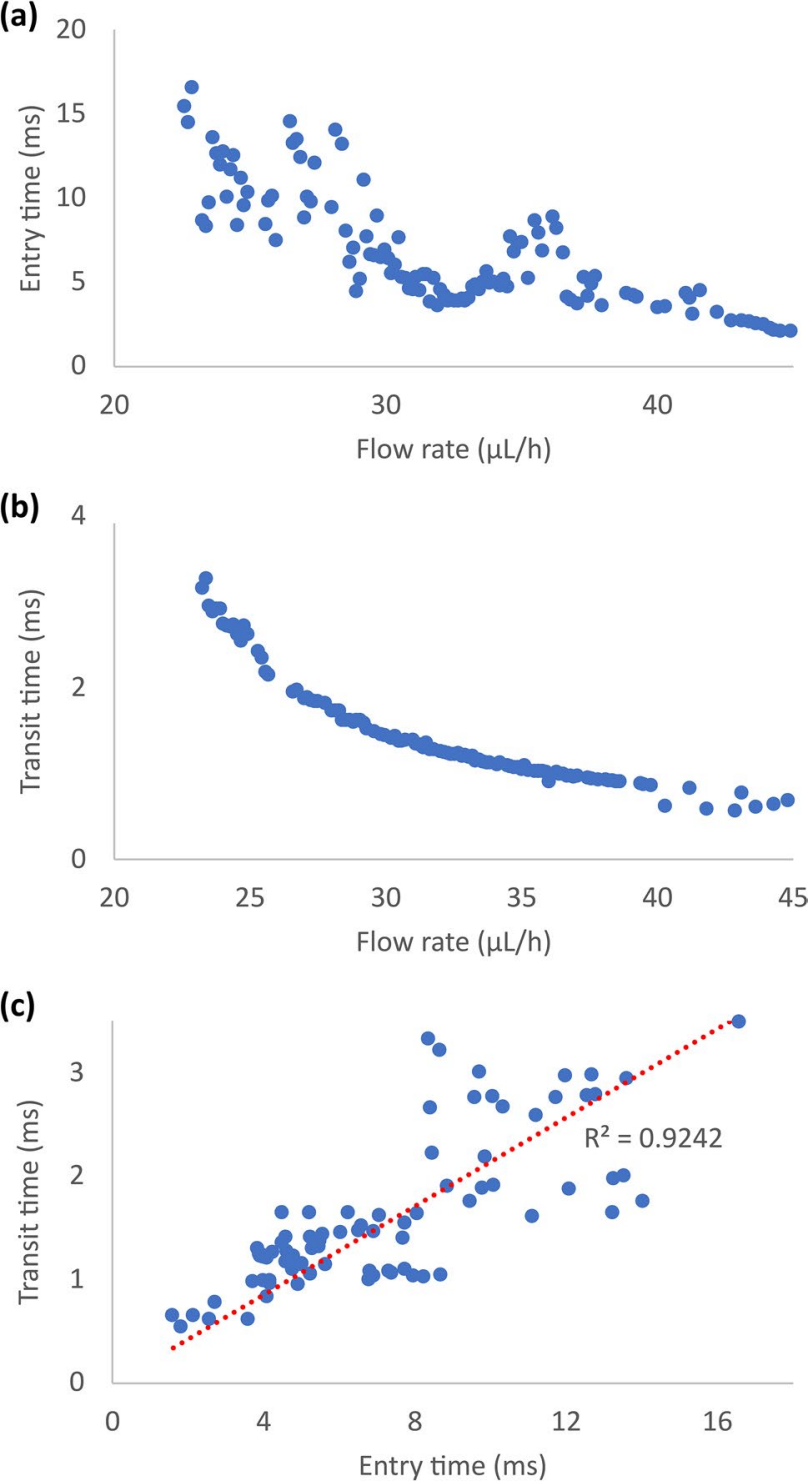
Effects of flow rate on the calculated entry time (**a**) and transit time (**b**) of a lung cancer cell in a microfluidic constriction as well as relationship between entry time and transit time at different flow rates (**c**). Simulations were performed using the validated spring-network model of the cell with accurately identified parameters. Both entry time (**a**) and transit time (**b**) generally follow a power law decrease with flow rate and they have a linear relationship with each other as the flow rate changes (**c**).

An immediate application of the proposed method with the accurately identified parameters of the spring-network model of the cancer cell is to predict the cell deformation and passage in the microchannel constriction at flow rates not measured experimentally. In the absence of experimental data to identify the friction coefficient for flow rates other than the two flow rates, a linear relationship between the friction coefficient and flow rate was assumed using ${\mu }_{f}^{1}$ and ${\mu }_{f}^{2}$ in Table 7. Figure **8**a,b illustrate the entry time and transit time of the cell as functions of flow rate, respectively. As Fig. **8**a shows, the cell entry time to the narrow constriction generally follows a power law decreasing trend with increasing the flow rate from 15.5 ms at 23 µL/h to 1.6 ms at 46 µL/h. Similarly, the general trend of cancer-cell transit time in the narrow constriction is a power law decrease with increasing the flow rate from 3.2 ms at 23 µL/h to 0.6 ms at 46 µL/h (Fig. **8**b). Interestingly, as Fig. **8**c shows, the transit time and entry time have a linear relationship with a coefficient of determination of 0.92. In this figure the increasing directions of both axes correspond to decreasing the flow rate as Fig. **8**a,b show. The values of the flow rate, entry time and transit time reported in Fig. **8**a,b are important for designing new experiments for obtaining cell-deformation information at several flow rates for further improving the accuracy of the identification of the parameters of the spring-network model of cancer cells.

Cell deformability was shown to be a promising label-free biomarker for cancer diagnosis^43^ and some high-throughput single-cell microfluidic devices have been proposed for cell characterization based on this biomarker^7,44,45^. Moreover, recently artificial intelligence was used to substantially increase the specificity of deformability-based cell sorting in microfluidic chips^46^. The method proposed in the present study can be used to obtain validated spring-network models of motion and deformation of cells that can be used for simulating passage of cells in microfluidic devices. This enables simulation of the cell deformation for designing new microfluidic devices by changing and optimizing the geometrical and fluid flow parameters before manufacturing the chip for cell characterization based on deformability.

Imaging modalities such as magnetic resonance phase contrast angiography in combination with mathematical optimization can be used for obtaining patient-specific vascular networks^47^. The validated cancer cell models also enable computationally efficient simulations of the metastatic processes in such vascular networks. These models can include cell-wall friction that can affect CTC adhesion and extravasation.

A sufficient model of deformation and motion of cancer cells should include viscosity of the cell as we demonstrated that ignoring the viscosity of the lung cancer cell had remarkable effects on its motion and deformation in the constriction. At the low flow rate (22.8 *µL/h*), the membrane viscosity encouraged deformation so that the cell completely squeezed in the microchannel sooner than it did in the absence of viscosity. However, at the high flow rate (45.6 *µL/h*), the cell viscosity resisted cell deformation and slowed down the deformation and therefore the entry time with viscosity was longer than it was without viscosity. This shows the sophisticated effects of the cell-membrane viscosity on the deformation and motion of the cancer cell and that these effects cannot be numerically achieved with a simplistic model.

A sufficient model of a cancer cell passing through a constriction should also consider the friction between the cell and the microfluidic wall as we demonstrated in this study. We showed that for a spring-network model, the flow-rate dependent friction coefficient should be identified and used in the model to accurately simulate the interactions of the cancer cell with the microchannel wall.

The method proposed in this study, the validated model and the knowledge obtained from it can be the basis for models of CTC in circulation, entrapment and extravasation stages of metastasis. Such models are important for obtaining better fundamental understandings of the metastasis process as well as for developing novel predictive tools.

### Limitations

Higher accuracy in the simulation may be attained if the model parameters are obtained for the cell with a higher number of nodes. However, this would increase the computational cost to the extent that it cannot be possible for the GA code to find an accurate solution for the model parameters based on the experimentally measured passage time. The algorithm proposed in this work should be improved to enable more computationally efficient parameter identification process for finer discretization.

Furthermore, as it has been shown for parameter identification of the RBC using the stretch experimental data, the GA code can efficiently and accurately identify model parameters when the number of inputs and outputs are increased. For cancer cell deformation in a microchannel, measuring the output (e.g., entry time) at several flow rates can increase the number of input–output pairs and therefore can enable more efficient and more accurate identification of the cancer-cell model parameters compared to what we reported here. Future experiments should be carried out considering this requirement.

In this study only one lung cancer cell line (H1975) was investigated. Future studied should consider other cell types and the performance of the proposed method for those cell types should be investigated.

This study only considered flow rates of up to 45.6 µL/h. Future experimental and modelling studies should consider the effects of higher flow rates on the modes of motion and deformation of the cell in the constriction and should investigate whether friction and membrane viscosity parameters still play crucial roles at such high flow rates.

For the two flow rates for which we had experimental data, the discrete friction coefficient was found to strongly depend on the flow rate. To correctly capture the physics of the interaction of the cell with the channel wall, in terms of the cell transit time, the friction coefficient was found to decrease from 0.0118 at 22.8 µL/h to 0.0009 at 45.6 µL/h. We observed that, compared to the low flow rate, at the high flow rate the cell nodes got closer to the channel wall causing a higher nodal normal repulsive force. The proposed algorithm balanced the intense normal repulsive force with a reduction in the coefficient of friction at the high flow rate (Eq. 15). These observations, however, were based on the experimental data at only two flow rates and should be verified by collecting experimental data at multiple flow rates and performing the parameter identification using the method proposed in this work.

## Data Availability

ESPResSo is an open-source code available from http://espressomd.org. Object-in-Fluid module is an open-source code available at https://github.com/icimrak/espresso/tree/python. The parameter identification code is available upon reasonable request from the correspondence author.
